# Some Further Perversion of Facts

**Published:** 1896-08

**Authors:** 


SOME FURTHER (POLITELY CALLED) "PERVERSION OF FACTS." The *Texas Medical News*, alias the "Windmill," late *Texas Sanitarian*, says that Dr. Daniel feels called upon to criticise every good act of the State Association, or words to that effect. We challenge any one to find within the pages of the JOURNAL during the time since Dr. Daniel's resignation as secretary one single word of criticism of, or comment, other than favorable, upon anything whatever the Association has done. He has called attention time and again to the fact that too much money was being paid for the transactions, and that the work could be done and had been done, under his administration, for much less money, and in a manner not only satisfactory, but such as to elicit commendation by the medical press generally. The transactions, during Dr. Daniel's service of six years, was pronounced "the handsomest volume of transactions gotten out by any State Association." We cite that of 1886, containing 792 pages, which cost only $1.55 per volume, while later, one of some 400 pages cost $1.56. Not a word of criticism has the JOURNAL contained upon anything the Association has done. Confine yourself to facts, brother "windmill."

